# Sequence signatures within the genome of SARS-CoV-2 can be used to predict host source

**DOI:** 10.1128/spectrum.03584-23

**Published:** 2024-03-04

**Authors:** Josip Rudar, Peter Kruczkiewicz, Oksana Vernygora, G. Brian Golding, Mehrdad Hajibabaei, Oliver Lung

**Affiliations:** 1 National Centre for Foreign Animal Disease, Canadian Food Inspection Agency, Winnipeg, Manitoba, Canada; 2 Department of Integrative Biology & Centre for Biodiversity Genomics, University of Guelph, Guelph, Ontario, Canada; 3 Department of Biology, McMaster University, Hamilton, Ontario, Canada; 4 Department of Biological Sciences, University of Manitoba, Winnipeg, Manitoba, Canada; Universidade Federal do Rio de Janeiro, Rio de Janeiro, Brazil

**Keywords:** machine learning, COVID-19, viral host adaptation, selection pressure, metric learning, biomarker discovery

## Abstract

We conducted an *in silico* analysis to better understand the potential factors impacting host adaptation of severe acute respiratory syndrome coronavirus 2 (SARS-CoV-2) in white-tailed deer, humans, and mink due to the strong evidence of sustained transmission within these hosts. Classification models trained on single nucleotide and amino acid differences between samples effectively identified white-tailed deer-, human-, and mink-derived SARS-CoV-2. For example, the balanced accuracy score of Extremely Randomized Trees classifiers was 0.984 ± 0.006. Eighty-eight commonly identified predictive mutations are found at sites under strong positive and negative selective pressure. A large fraction of sites under selection (86.9%) or identified by machine learning (87.1%) are found in genes other than the spike. Some locations encoded by these gene regions are predicted to be B- and T-cell epitopes or are implicated in modulating the immune response suggesting that host adaptation may involve the evasion of the host immune system, modulation of the class-I major-histocompatibility complex, and the diminished recognition of immune epitopes by CD8+ T cells. Our selection and machine learning analysis also identified that silent mutations, such as C7303T and C9430T, play an important role in discriminating deer-derived samples across multiple clades. Finally, our investigation into the origin of the B.1.641 lineage from white-tailed deer in Canada discovered an additional human sequence from Michigan related to the B.1.641 lineage sampled near the emergence of this lineage. These findings demonstrate that machine-learning approaches can be used in combination with evolutionary genomics to identify factors possibly involved in the cross-species transmission of viruses and the emergence of novel viral lineages.

**IMPORTANCE:**

Severe acute respiratory syndrome coronavirus 2 (SARS-CoV-2) is a highly transmissible virus capable of infecting and establishing itself in human and wildlife populations, such as white-tailed deer. This fact highlights the importance of developing novel ways to identify genetic factors that contribute to its spread and adaptation to new host species. This is especially important since these populations can serve as reservoirs that potentially facilitate the re-introduction of new variants into human populations. In this study, we apply machine learning and phylogenetic methods to uncover biomarkers of SARS-CoV-2 adaptation in mink and white-tailed deer. We find evidence demonstrating that both non-synonymous and silent mutations can be used to differentiate animal-derived sequences from human-derived ones and each other. This evidence also suggests that host adaptation involves the evasion of the immune system and the suppression of antigen presentation. Finally, the methods developed here are general and can be used to investigate host adaptation in viruses other than SARS-CoV-2.

## INTRODUCTION

The mutation spectrum of the severe acute respiratory syndrome coronavirus 2 (SARS-CoV-2) could serve as a useful tool to monitor the evolution of the virus. For example, it has been used as a key piece of evidence to identify mice as the potential origin of the Omicron variant of the virus (1). In addition, we have previously used this approach to provide evidence suggesting that SARS-CoV-2 found in deer was evolving in parallel to that found spreading in human populations (2). However, apart from work providing evidence for changes in the mutation spectrum being linked to host factors, such as the activity of RNA editing enzymes, relatively little has been done to probe the use cases and limits of the mutation spectrum and other genomic information about SARS-CoV-2. In this work, we add to the current literature by investigating if the mutation spectrum and other genomic information, such as single nucleotide variants (SNVs) and amino acid changes, can be used in conjunction with machine learning to train models capable of predicting host species of origin.

Due to the broad host tropism of SARS-CoV-2 and evidence suggesting it could form reservoirs in wild animal populations (1–3), the recent COVID-19 pandemic has underscored the need to identify factors involved in host adaptation and spillover into new susceptible hosts. Because adaptation to a new host imposes strong evolutionary pressure, successful transmission and establishment in a new host type are driven by the natural selection forces, both diversifying and purifying. Positive (diversifying) selection promotes the emergence of new variants that may be more successful at establishing in a new host while purifying selection purges deleterious mutations that disrupt protein functionality. It has been shown that negative selection plays an important role in the virus-host evolution by preserving the genomic structure and organization of viruses (4). However, even at sites under strong negative selection, signatures of the host switch can be found when a silent mutation variant becomes fixed after a transmission bottleneck event (5). This is important since recent work has shown the evolutionary trajectory of SARS-CoV-2 in alternative hosts could have important health ramifications in human populations (1, 2, 6, 7). If predictive models of host type prove to be useful, then recent advances in explainable artificial intelligence could be applied to narrow the list of factors involved in the cross-species transmission of SARS-CoV-2 (8). There is some precedence for this work since these approaches have been used to identify codons in the spike protein associated with different SARS-CoV-2 variants (8). In this work, we introduce an improved feature selection algorithm, Triglav, which takes advantage of these developments to identify genomic changes associated with spillover into new animal hosts. We also apply TreeOrdination, an unsupervised learning algorithm based on decision tree ensembles, to identify new sequences related to the evolution of the recently described B.1.641 lineage, which was shown to have potential for deer-to-human transmission (2, 9).

## MATERIALS AND METHODS

### Data set

The human sequences used in this study were from North America and can be found in Table S9 by Pickering et al. (2). We also downloaded all complete and high-coverage North American white-tailed deer (*Odocoileus virginianus*) and mink (*Neovison vison*) sequences present in the GISAID database as of 17 February 2022 using the GISAIDR (version 3) (10). The total number of sequences downloaded was 710 with 203 belonging to mink and 507 belonging to white-tailed deer. Nucleotide and amino acid mutations, insertions, and deletions were identified using Nextclade (https://clades.nextstrain.org) (11). This analysis was restricted to North American human-, mink-, and white-tailed deer-derived samples for several reasons. First of all, wild *Odocoileus virginianus* populations are only found in North America, and along with *Neovison vison* and humans, sustained transmission of SARS-CoV-2 was observed in these species (2, 12). Furthermore, SARS-CoV-2 is likely to have spilled over into these hosts from nearby human populations. These animals also represent important sources of food and are economically important in certain regions of the United States of America and Canada. For these reasons, we believe that the inclusion of sequences from outside North America could introduce noise into this analysis potentially obfuscating the discovery of adaptive mutations in white-tailed deer and North American mink hosts. We also removed sequences that came from dead-end hosts since these hosts are unlikely to contribute to the epidemiology of SARS-CoV-2. Training machine learning models using these sequences can be problematic since they tend to be very similar to human sequences due to the lack of sustained transmission (13, 14). Finally, sequences isolated from poorly represented hosts were removed since the inclusion of these sequences could potentially reduce the robustness of machine learning models.

To create the mutation spectrum, the Nextclade nucleotide mutation information occurrence counts and frequencies were computed for each element of the spectrum (C > U, G > A, etc.) with respect to the reference genome Wuhan-Hu-1 (MN908947.3) (1). Only samples with at least 10 mutations were retained for further analysis. In addition, a second data set was created using SNVs, structural variants (SV), and amino acid changes as features. Sequences with at least 10 mutations were retained. In addition, for a SNP, SV, or amino acid change to be retained, it must be present in at least five samples. The mutation spectrum and mutation data sets were then used for all supervised and unsupervised analyses. Filtering of sequences using the mutation spectrum was done to remove samples that were not significantly different from the reference genome. SNVs, SVs, and amino acid mutations were filtered to remove features with poor predictive value due to their presence in only a few sequences. A simplified overview of the filtering steps taken is outlined in Fig. S1. While we recognize that filtering in this manner could remove important information, filtering out rare features does have certain advantages. These actions tend to preserve the overall structure of the data and improve model accuracy and sensitivity (15). Basic filtering steps, such as those outlined here and in Fig. S1, are also important since they reduce the amount of noise caused by the variability in how samples from public databases are processed, sequenced, and analyzed. Finally, the goal of this work is to identify potential global markers of host adaptation. Rare changes, by definition, would fall outside the scope of this goal.

### Training of supervised models

The mutation spectrum and mutation data sets were used to train an Extra Trees Classifier (ETC), a LANDMark classifier (version 2.1.0), Linear Support Vector Machine Classifier (LSVM), a Logistic Regression (LR) Classifier, and a K-Nearest Neighbors (KNN) Classifier (16, 17). Other than LANDMark, a tree-based ensemble that uses multivariate splits at the internal nodes of each tree, scikit-learn (version 1.2.2) provided the machine learning models used in this work (18).

ETC is a decision-tree ensemble classifier that aggregates the results of multiple decision trees to reduce the variance in outcome prediction (16). ETC can model complex non-linear relationships and is more robust to noise when trained on high-dimensional data (16). Unlike the Random Forest Classifier, the improvement in generalization performance can be attributed to the way the ETC randomizes the cut points within each internal node (16, 19). All ETC classifiers were trained using 512 trees.

The LANDMark classifier is also a decision-tree ensemble. Unlike the trees used to construct an ETC, the internal nodes of LANDMark decision trees select the best possible classification model to create a binary split (17). Although this increases the overall training time significantly, this change allows LANDMark decision trees to adapt to the local geometry of the data (17). LANDMark classifiers were trained without the use of neural networks or models that use L1-regularization to improve the training time. Zero variance features were identified and removed before each tree was grown (18). Both ETC and LANDMark models allowed each tree to increase to its maximum depth. Finally, LANDMark models were constructed with and without the use of a random linear oracle (20).

LSVM models map each observation into a higher dimensional representation to find an optimal separating hyperplane between different classes of samples (21). The choice for the regularization parameter, *C*, was selected using a cross-validated grid search from eight logarithmically spaced values within the range of 0.0001 to 1000 (18).

KNN classifiers are widely used due to their simplicity and ease of use. These classifiers work by finding the *K* nearest neighbors to an unknown sample, where *K* is the number of neighbors. Once these neighbors are identified, classification simply involves outputting the label of the majority class within the neighborhood of the unknown samples (22). Scikit-learn was used to conduct a cross-validated grid search to find the best value of *K* (from 3, 5, 7, or 9) and the optimal weighting method (18).

Finally, LR is a statistical approach that models the probability of an output (a target class) using a linear combination of the input features (23). L2 regularization was used to reduce the possibility of overfitting. The regularization parameter, *C*, was selected from logarithmically spaced values between 1 × 10^−4^ and 1 × 10^4^ using stratified cross-validation (18).

To ensure that enough data were present, only clades containing a minimum of 20 samples were used for the supervised analysis. This data set contains 497 deer, 5,987 human, and 203 mink sequences and 17,760 features. Of these, 4,095 were retained after filtering. In addition, since Nextstrain clades 20C and 21J contained the largest number of samples from deer and mink hosts, a separate supervised analysis was performed using these clades. The subset belonging to clade 20C contains 38 deer, 4,423 human, and 125 mink sequences. Filtering resulted in the retention of 1,910 out of 10,128 features in this data set. The subset corresponding to clade 21J contains 251 deer and 122 human samples. Of the 3,909 features in this data set, 479 remained after filtering. Each data set was split into testing and training data sets using fivefold stratified cross-validation with five repeats, and generalization performance was measured using the balanced accuracy score (18). Random under-sampling of human samples was performed on data sets containing data from the “All Clades” and clade 20C data sets to minimize the impact of human sample over-representation. Three hundred human, 90 mink, and 28 deer samples were randomly selected on each fold when training using data from clade 20C while 600 human, 150 mink, and 375 deer samples were randomly selected when training on data from “All Clades.” “baycomp” (version 1.0.2) (24) was then used to assess the equivalence in balanced accuracy scores generated from each model with the region of practical equivalence set to 0.05. The entire analysis was conducted in Python 3.11.

### Identification of discriminative features using feature clustering

Triglav (version 1.0.3) (https://github.com/jrudar/Triglav), a wrapper approach to feature selection, was used to identify potentially important genetic features for discriminating between viruses isolated from different hosts (25). Briefly, the Triglav algorithm draws inspiration from the Boruta feature selection approach to identify a stable set of features. Features are first clustered together, and a subset of features from each cluster is selected. These are used to create a randomized data set from the marginal distribution of each of the selected features. These original data are extended by the randomized counterpart, and this extended data set is used to train an Extremely Randomized Trees classifier (16, 26, 27). Following this, Shapley values are calculated to summarize the impact of each original and randomized feature on the output of the model (28). This process is repeated several times to generate a distribution of impact scores associated with each cluster. A Wilcoxon rank-sum test is then used to identify the most impactful clusters of features (those with impact scores greater than their permuted counterparts), resulting in a hit or a rejection for each cluster. Beta-binomial distributions are then used to select and reject feature clusters with significant hit and rejection rates, respectively.

The mutation data set was processed and analyzed with Triglav. Dissimilarities between features were measured using the unsupervised Extremely Randomized Trees approach (29). A dendrogram was created using complete-linkage clustering and visually examined to create a flat clustering to estimate a distance threshold (Fig. S2). A distance threshold of 0.5 was manually chosen to identify impactful clusters containing related features with Triglav. Global feature importance scores were calculated, and the number of iterations was set to 60. Finally, a Logistic Regression classifier was used as the classification model since this model can be used to identify a linear decision boundary between hosts. Triglav’s “alpha” parameter, which controls when clusters are rejected during the Wilcoxon signed-rank test and the beta-binomial tests, was set to 0.01. All remaining parameters remained at the default setting. Repeated Stratified K-Fold cross-validation (5 splits, 20 repeats) was used to create 100 different Triglav models. The features identified by each model were then saved for additional analysis.

The best set of mutations was first identified by calculating both the balanced accuracy and F1-Score of each set of mutations using Triglav and removing samples with host-province or host-province-clade combinations with a frequency of 10 or less. Stratified random sampling based on host species origin and geographic origin (and clade for the “All Clades” data set) was used to split the samples from each data set into training (80%) and testing (20%) data. Since the training data for the “All Clades” and clade 20C data sets contained many more human samples than animal samples, a representative subset of 600 (All Clades) and 300 (clade 20C) human training samples were selected based on their clade and/or geographic origin. Two LR models were trained with the training data after subsetting using the selected and rejected features. The set of selected features and the training/testing set associated with the best balanced accuracy score were used to identify the 20 most predictive features using SAGE (30). This approach to explainable artificial intelligence quantifies a feature’s importance using the game theory. A Bayesian *t*-test was then used to assess the equivalence in balanced accuracy scores generated from each model (24). For this analysis, the region of practical equivalence was set to 0.05. Finally, average linkage hierarchical clustering of samples and features using the Hamming distance was applied to both samples and Triglav-selected features to visualize any patterns of mutations associated with the host source of each sample (31, 32).

### Identification of sites under positive and negative selection

To identify protein sites experiencing selection pressure, we used the fast unconstrained Bayesian approximation (FUBAR) and single-likelihood ancestor counting (SLAC) methods (33, 34). Selection inference analysis was performed using nucleotide sequence alignments of each major gene in the “All Clades” data set. The input guidance gene trees were generated using the maximum-likelihood method as implemented in FastTree v.2.1 under the general time-reversible (GTR) + CAT model and otherwise default settings (35). FUBAR and SLAC analyses for the large “All Clades” data set were performed using HyPhy v.2.2.4 (36, 37). Protein sites were inferred to be under selection if the posterior probability of inferred selection pressure (positive or negative) was ≥0.9 (FUBAR) or with a *P* value of less than 0.1 (SLAC).

In addition to searching for positions under positive and negative selection, Triglav identified different sets of predictive SNVs, SVs, and amino acid changes due to 100 different subsets of data being used to train the algorithm. Frequently selected changes were identified using a binomial test with an *n* of 100 and a *P* of 0.65. The number of successes, *k*, was the number of times a SNV, SV, or amino acid change was selected by Triglav. The Benjamini-Hochberg adjustment for multiple comparisons was then applied, and only those mutations with a significance of less than or equal to 0.05 were retained for further analysis. A multiple sequence alignment (MSA) of the “All Clades” subset used for feature clustering was constructed using MAFFT (v.7.490) and used to infer a maximum-likelihood time-tree with IQ-TREE (v2.2.0) using the GTR model (38, 39). The overlap between sites under positive and negative selection and those consistently selected by Triglav were also identified. A bi-clustering using the phylogeny and the overlap in features was then built using “ggtree” in R. The heatmap created by this clustering visualizes the changes that occur in each gene and clusters of changes associated with each clade in the phylogeny (2, 32). A separate figure was created to visualize sites under selective pressure that were not identified by Triglav.

### Identification of potential spillover events

We investigated the ability of mutation information and the mutation spectra to detect sequences related to a recently identified potential deer-to-human spillover event within a unique lineage (B.1.641) identified in Ontario white-tailed deer. This analysis was conducted using a recently described approach known as TreeOrdination (version 1.3.3) (2, 9). Briefly, this approach builds a high-dimensional representation of the data set using multivariate decision trees, which have been shown to effectively partition the original input space. This high-dimensional embedding is then used to create a lower dimensional projection analogous to a principal components analysis. Human-, mink-, and deer-derived SARS-CoV-2 sequences from clade 20C lineage B.1, the parent lineage of B.1.641 consisting of samples derived from a potential deer-to-human zooanthroponotic transmission event (2), were selected for the TreeOrdination analysis since the evolutionary history of this lineage is unclear and the identification of related sequences may improve our understanding of how cross-species transmission of SARS-CoV-2 occurs. The human-derived sequence from this event, hCoV-19/Canada/ON-PHL-21–44225/2021, was removed from the data set for use as a query to conduct a nearest neighbor search using TreeOrdination projected data. Twenty different TreeOrdination iterations were run to construct a high-dimensional embedding. The Uniform Manifold Approximation and Projection approach using the Hamming distance was then used to transform this embedding into a lower 15-dimensional projection (40). There were 400 human, 125 mink, and 38 deer sequences randomly selected during each TreeOrdination iteration to minimize the impact of class imbalance. At each iteration, 128 LANDMark estimators with the neural network functionality disabled were constructed. If an Extremely Randomized Tree classifier was selected at a node in a LANDMark tree, it was allowed to be grown to a maximum depth of 8. The 50 nearest neighbors to the query were identified using the “NearestNeighbors” function from scikit-learn (18). A maximum-likelihood phylogenetic time tree was inferred from a MAFFT (v.7.490) MSA using the sequences identified in the search with IQ-TREE (v2.2.0) using the GTR MODEL (38, 39). Amino acid mutation profiles were visualized alongside the phylogenetic tree using the “ggtree” R package (2, 32). A TreeOrdination analysis was also conducted using the mutational spectra to determine if the results using the spectra were similar to those from the mutation information. TreeOrdination automatically transformed the mutation counts using an appropriate transformation function to avoid conflicting with the generation of properly randomized data.

## RESULTS

### Predictive models for host type can be created using the mutation spectrum

Developing predictive models for SARS-CoV-2 host species of origin can provide valuable insights into how the virus evolves after a spillover event and identify potential spill-over events. We successfully predicted host species of origin using the mutation spectrum while benchmarking a variety of models and approaches (Table 1). LANDMark and ETC models resulted in the best predictive performance when all clades were included in the analysis. The cross-validated balanced accuracy scores of these models were 0.918 ± 0.019 and 0.920 ± 0.017, respectively, and the Bayesian *t*-test results suggest that the probability of a difference in the performance of these models is also negligible. The probability that other models (KNN, LSVM, and LR) outperformed ETC and LANDMark was also very low. When considering the mutation information data, the generalization performance increased substantially and all models performed similarly and achieved a balanced accuracy score of at least 0.95 (Table 2). When each clade was considered separately and when only trained on the mutation spectrum data, the ETC, KNN, and LANDMark classifiers performed best. The Bayesian *t*-test results also suggest that these three models will perform equivalently when trained using this data. In contrast, all classifiers other than K-Nearest Neighbors will likely have similar generalization performance when trained using the mutation information data.

**TABLE 1:** Pairwise comparisons between the generalization performance of models trained using only the mutation spectrum data^*a*^

	Model A	Model B	Mean A	Mean B	*P*(A > B)	*P*(A == B)	*P*(A < B)
*Clade 20C*	LANDMark	Extra Trees	0.745 ± 0.061	0.773 ± 0.055	0	0.882	0.118
	LANDMark	Logistic Regression	0.745 ± 0.061	0.621 ± 0.040	0.950	0.05	0
	LANDMark	K-Nearest Neighbors	0.745 ± 0.061	0.759 ± 0.060	0.035	0.822	0.143
	LANDMark	Linear SVC	0.745 ± 0.061	0.618 ± 0.034	0.973	0.027	0
	Extra Trees	Logistic Regression	0.773 ± 0.055	0.621 ± 0.040	0.991	0.009	0
	Extra Trees	K-Nearest Neighbors	0.773 ± 0.055	0.759 ± 0.060	0.135	0.840	0.025
	Extra Trees	Linear SVC	0.773 ± 0.055	0.618 ± 0.034	0.997	0.003	0
	Logistic Regression	K-Nearest Neighbors	0.621 ± 0.040	0.759 ± 0.060	0.0	0.015	0.985
	Logistic Regression	Linear SVC	0.621 ± 0.040	0.618 ± 0.034	0.031	0.950	0.019
	K-Nearest Neighbors	Linear SVC	0.759 ± 0.060	0.618 ± 0.034	0.989	0.011	0
*Clade 21J*	LANDMark	Extra Trees	0.779 ± 0.056	0.777 ± 0.060	0.002	0.996	0.001
	LANDMark	Logistic Regression	0.779 ± 0.056	0.774 ± 0.057	0.027	0.963	0.010
	LANDMark	K-Nearest Neighbors	0.779 ± 0.056	0.745 ± 0.059	0.282	0.715	0.003
	LANDMark	Linear SVC	0.779 ± 0.056	0.770 ± 0.054	0.036	0.959	0.006
	Extra Trees	Logistic Regression	0.777 ± 0.060	0.774 ± 0.057	0.019	0.970	0.011
	Extra Trees	K-Nearest Neighbors	0.704 ± 0.059	0.745 ± 0.059	0.261	0.734	0.005
	Extra Trees	Linear SVC	0.704 ± 0.059	0.770 ± 0.054	0.042	0.945	0.013
	Logistic Regression	K-Nearest Neighbors	0.774 ± 0.057	0.745 ± 0.059	0.204	0.794	0.003
	Logistic Regression	Linear SVC	0.333 ± 0.0	0.770 ± 0.054	0	1	0
	K-Nearest Neighbors	Linear SVC	0.745 ± 0.059	0.770 ± 0.054	0.003	0.847	0.150
*All Clades*	LANDMark	Extra Trees	0.918 ± 0.019	0.920 ± 0.017	0	1	0
	LANDMark	Logistic Regression	0.918 ± 0.019	0.733 ± 0.035	1	0	0
	LANDMark	K-Nearest Neighbors	0.918 ± 0.019	0.891 ± 0.016	0.014	0.0986	0
	LANDMark	Linear SVC	0.918 ± 0.019	0.738 ± 0.038	1	0	0
	Extra Trees	Logistic Regression	0.920 ± 0.017	0.733 ± 0.035	1	0	0
	Extra Trees	K-Nearest Neighbors	0.920 ± 0.017	0.891 ± 0.016	0.006	0.994	0
	Extra Trees	Linear SVC	0.920 ± 0.017	0.738 ± 0.038	1	0	0
	Logistic Regression	K-Nearest Neighbors	0.733 ± 0.035	0.891 ± 0.016	0	0	1
	Logistic Regression	Linear SVC	0.733 ± 0.035	0.738 ± 0.038	0	1	0
	K-Nearest Neighbors	Linear SVC	0.891 ± 0.016	0.738 ± 0.038	1	0	0

^*a*^ A Bayesian *t*-test was used to determine the probability that the balanced accuracy score of model A either exceeds, is lower, or is equivalent to the performance of model B. Feature selection using Triglav was not performed to generate these results.

**TABLE 2:** Pairwise comparison between the generalization performance of models trained using only the mutation data^*a*^

	Model A	Model B	Mean A	Mean B	*P*(A > B)	*P*(A == B)	*P*(A < B)
*Clade 20C*	LANDMark	Extra Trees	0.965 ± 0.035	0.968 ± 0.037	0.001	0.996	0.003
	LANDMark	Logistic Regression	0.965 ± 0.035	0.972 ± 0.035	0.001	0.990	0.009
	LANDMark	K-Nearest Neighbors	0.965 ± 0.035	0.875 ± 0.051	0.948	0.052	0
	LANDMark	Linear SVC	0.965 ± 0.035	0.973 ± 0.030	0	0.999	0.001
	Extra Trees	Logistic Regression	0.968 ± 0.037	0.972 ± 0.035	0.004	0.985	0.011
	Extra Trees	K-Nearest Neighbors	0.968 ± 0.037	0.875 ± 0.051	0.969	0.031	0
	Extra Trees	Linear SVC	0.968 ± 0.037	0.973 ± 0.030	0.003	0.985	0.012
	Logistic Regression	K-Nearest Neighbors	0.972 ± 0.035	0.875 ± 0.051	0.952	0.048	0
	Logistic Regression	Linear SVC	0.972 ± 0.035	0.973 ± 0.030	0	0.999	0.001
	K-Nearest Neighbors	Linear SVC	0.875 ± 0.051	0.973 ± 0.030	0	0.023	0.977
*Clade 21J*	LANDMark	Extra Trees	0.892 ± 0.044	0.896 ± 0.042	0	1	0
	LANDMark	Logistic Regression	0.892 ± 0.044	0.886 ± 0.045	0.002	0.998	0
	LANDMark	K-Nearest Neighbors	0.892 ± 0.044	0.764 ± 0.049	1	0	0
	LANDMark	Linear SVC	0.892 ± 0.044	0.882 ± 0.042	0.008	0.991	0
	Extra Trees	Logistic Regression	0.896 ± 0.042	0.886 ± 0.045	0.009	0.990	0
	Extra Trees	K-Nearest Neighbors	0.896 ± 0.042	0.764 ± 0.049	1	0	0
	Extra Trees	Linear SVC	0.896 ± 0.042	0.882 ± 0.042	0.023	0.976	0
	Logistic Regression	K-Nearest Neighbors	0.886 ± 0.045	0.764 ± 0.049	0.999	0.001	0
	Logistic Regression	Linear SVC	0.886 ± 0.045	0.882 ± 0.042	0.004	0.994	0.001
	K-Nearest Neighbors	Linear SVC	0.764 ± 0.049	0.882 ± 0.042	0	0.003	0.997
*All Clades*	LANDMark	Extra Trees	0.982 ± 0.007	0.982 ± 0.006	0	1	0
	LANDMark	Logistic Regression	0.982 ± 0.007	0.984 ± 0.005	0	1	0
	LANDMark	K-Nearest Neighbors	0.982 ± 0.007	0.950 ± 0.018	0.035	0.965	0
	LANDMark	Linear SVC	0.982 ± 0.007	0.985 ± 0.018	0.0	1	0.0
	Extra Trees	Logistic Regression	0.982 ± 0.006	0.984 ± 0.005	0.0	1	0.0
	Extra Trees	K-Nearest Neighbors	0.982 ± 0.006	0.950 ± 0.018	0.027	0.973	0
	Extra Trees	Linear SVC	0.982 ± 0.006	0.985 ± 0.018	0	1	0
	Logistic Regression	K-Nearest Neighbors	0.984 ± 0.005	0.950 ± 0.018	0.043	0.957	0
	Logistic Regression	Linear SVC	0.984 ± 0.005	0.985 ± 0.018	0	1	0
	K-Nearest Neighbors	Linear SVC	0.950 ± 0.018	0.985 ± 0.018	0	0.947	0.053

^*a*^ A Bayesian *t*-test was used to determine the probability that the balanced accuracy score of model A either exceeds, is lower, or is equivalent to the performance of model B. Feature selection using Triglav was not performed to generate these results.

We also observed that generalization performance did not increase substantially when combining both the mutation spectrum and mutation information data sets (Tables S3, S6, and S9). Finally, LANDMark has the functionality to randomly partition the input samples before testing classifiers at each internal node (a random oracle). In previous work, we determined that this improves generalization performance (17). However, enabling this parameter may not have been appropriate since the virus was introduced into animal populations through multiple independent introduction events (41). Since each introduction event can lead to the evolution of a different set of unique mutations, enabling this function could lead to a sub-optimal partitioning of samples (42). To test this, we re-ran the generalization performance tests with the random oracle disabled and found that generalization performance did not differ significantly when using this parameter (Tables S1 to S9). Therefore, while a considerable amount of information is present in the mutation spectrum, models trained on SNVs, SVs, and amino acid changes result in better generalization performance.

### Feature clustering can be used to identify host discriminatory markers within each clade

LR classifiers trained on Triglav-selected features identified had good generalization performance. Models trained on the selected features from clade 20C had a mean balanced accuracy score of 0.961 ± 0.049. In contrast, models trained on the removed features had a mean balanced accuracy score of 0.594 ± 0.089. A Bayesian *t*-test strongly suggested that training using rejected features did not result in better models with the Probability (Selected > Rejected) = 1. A similar result was obtained for clade 21J (mean balanced accuracy score of 0.898 ± 0.033). However, the set of rejected features still retained some predictive value (0.627 ± 0.073). Despite this, Triglav appears to discriminate between hosts well in both clades (Table S10). Although the rejected features from the “All Clades” data set had a high balanced accuracy score and generalization performance, the F1-score demonstrated that there is a significant drop in generalization performance when using these features (Table S10). This indicates that the model’s precision and/or recall in predicting one or more hosts is compromised when using rejected features.

**Fig 1:**
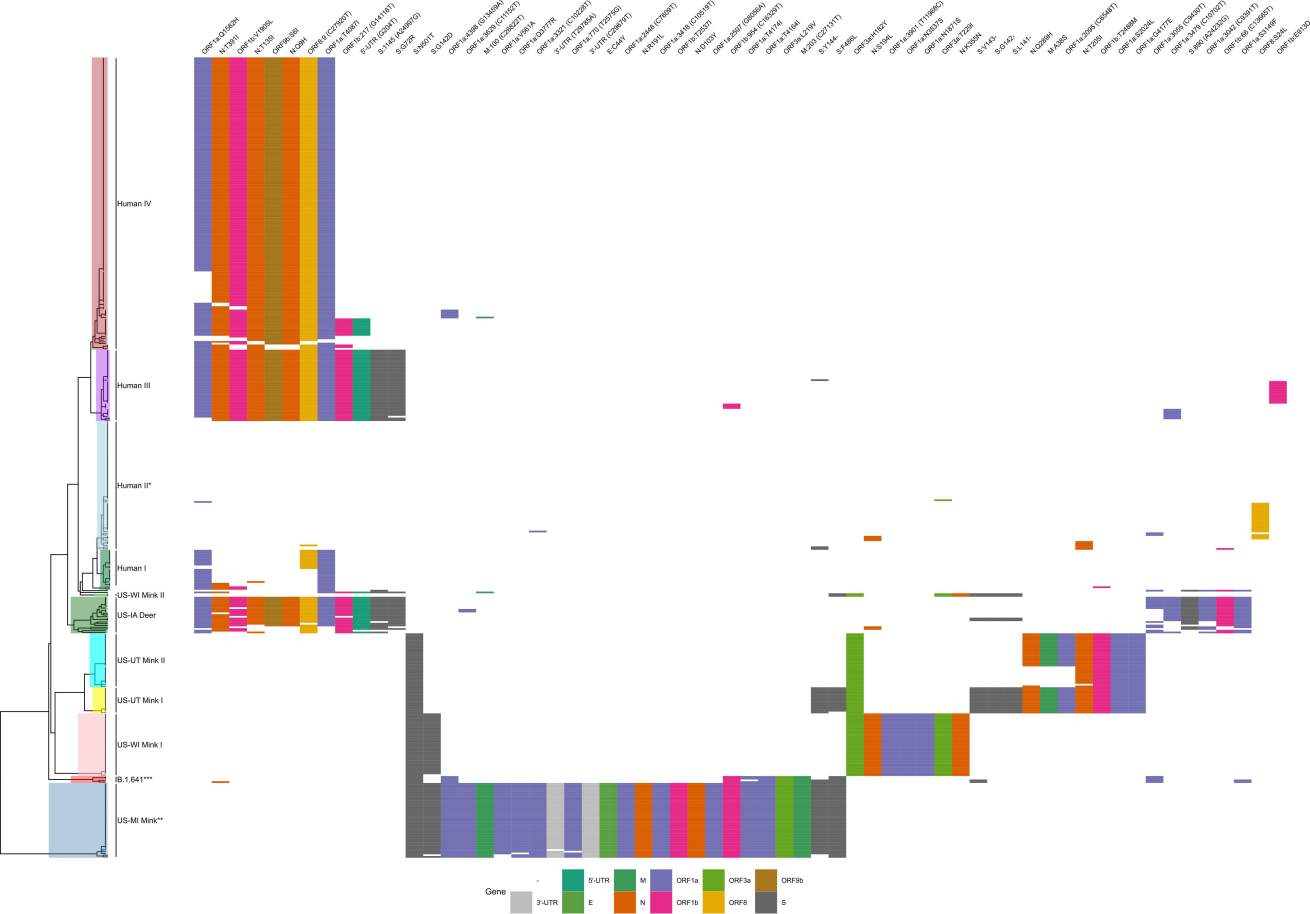
A bi-clustering of clade 20C. The bi-clustering shows the relationship between deer (*Odocoileus virginianus*), human (*Homo sapiens*), and mink (*Neovison vison*) sequences based on their genome-wide amino acid changes relative to the reference genome, Wuhan-Hu-1 (MN908947.3). Changes in each gene are colored differently. Rows (samples) and columns (features) were clustered separately using hierarchical clustering and average linkage with distances calculated using the Hamming metric. Groups marked with a single asterisk (*) indicate mostly human-derived sequences while groups marked with a double asterisk (**) indicate groups containing mostly animal-derived sequences. A triple asterisk (***) indicates the location of linear B.1.641 and related human-derived sequences from Michigan.

**Fig 2:**
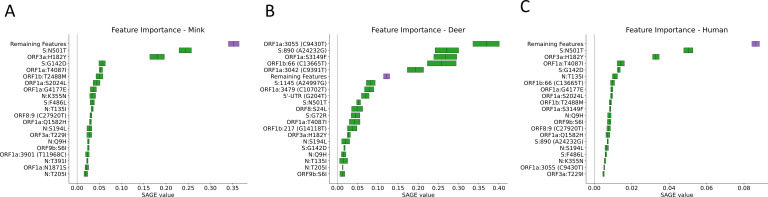
The top 20 most important features associated with each species from clade 20C. These scores are SAGE values, with higher values representing more important features. The collective importance of the remaining features is high, but unreported features found in this category have a SAGE value lower than the 20th-best actual feature. Each panel represents features important for distinguishing between mink (**A**), deer (**B**), and human (**C**) hosts. When interpreting the direction of a feature’s importance, the feature’s general presence or absence in the samples derived from the host should be determined.

**Fig 3:**
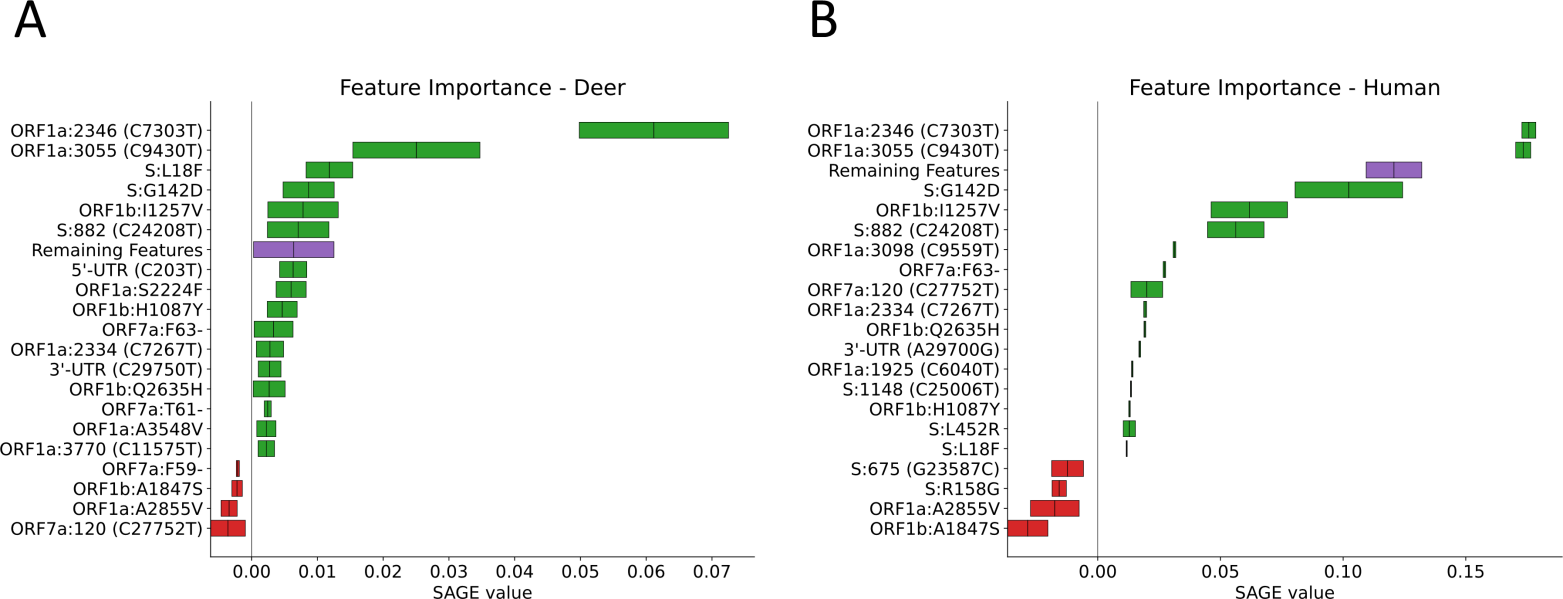
The top 20 most important features associated with each species from clade 21J. These scores are SAGE values, with higher values representing more important features. The collective importance of the remaining features is high, but unreported features found in this category have a SAGE value lower than the 20th-best actual feature. Each panel represents features important for distinguishing between deer (**A**) and human (**B**) hosts.

**Fig 4:**
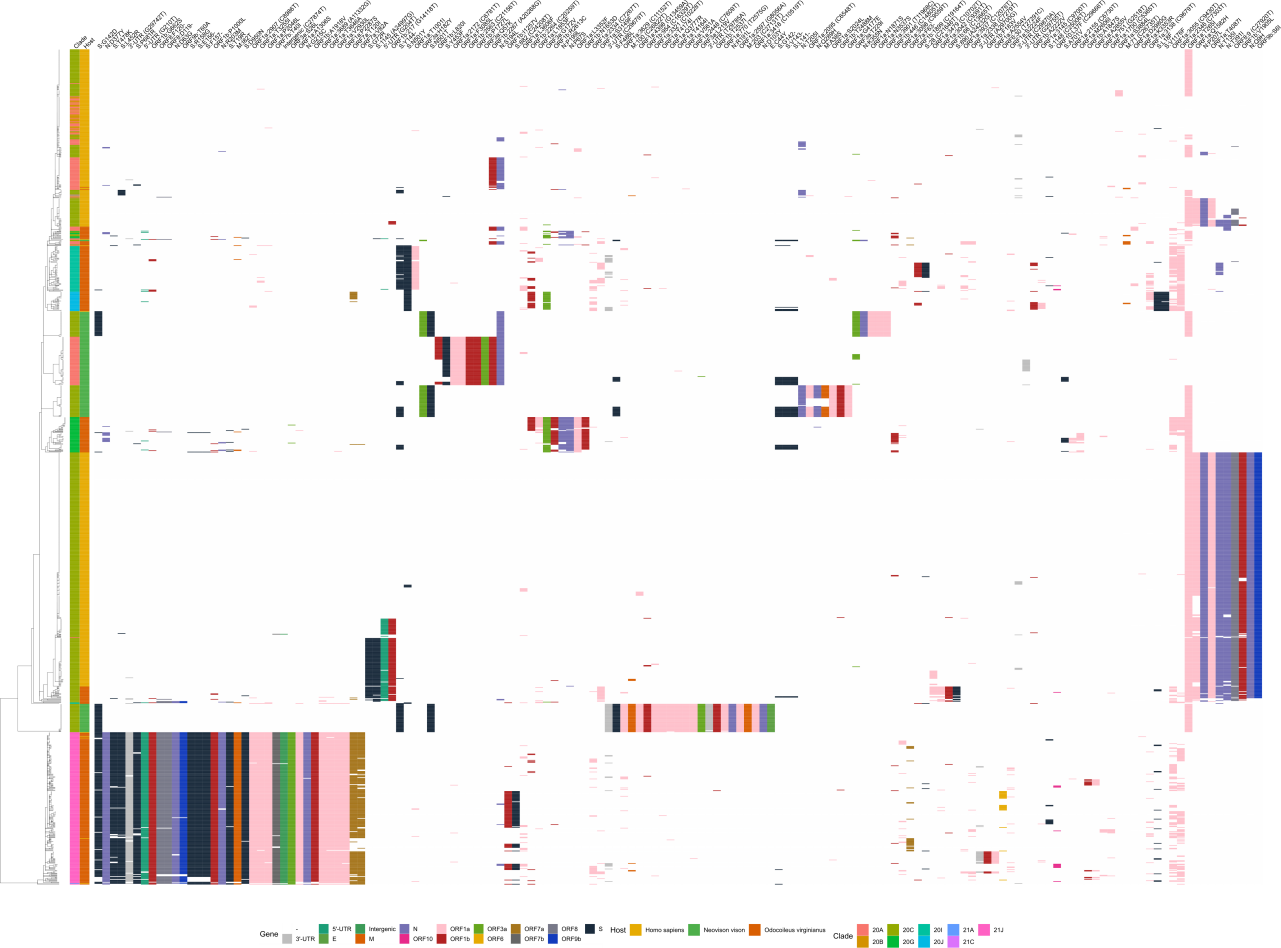
A bi-clustering of the “All Clades” data set. The bi-clustering shows the relationship between deer (*Odocoileus virginianus*), human (*Homo sapiens*), and mink (*Neovison vison*) sequences based on their genome-wide amino acid changes relative to the reference genome, Wuhan-Hu-1 (MN908947.3). Changes in each gene are colored differently. Rows (samples) and columns (features) were clustered separately using hierarchical clustering and average linkage with distances calculated using the Hamming metric.

**Fig 5:**
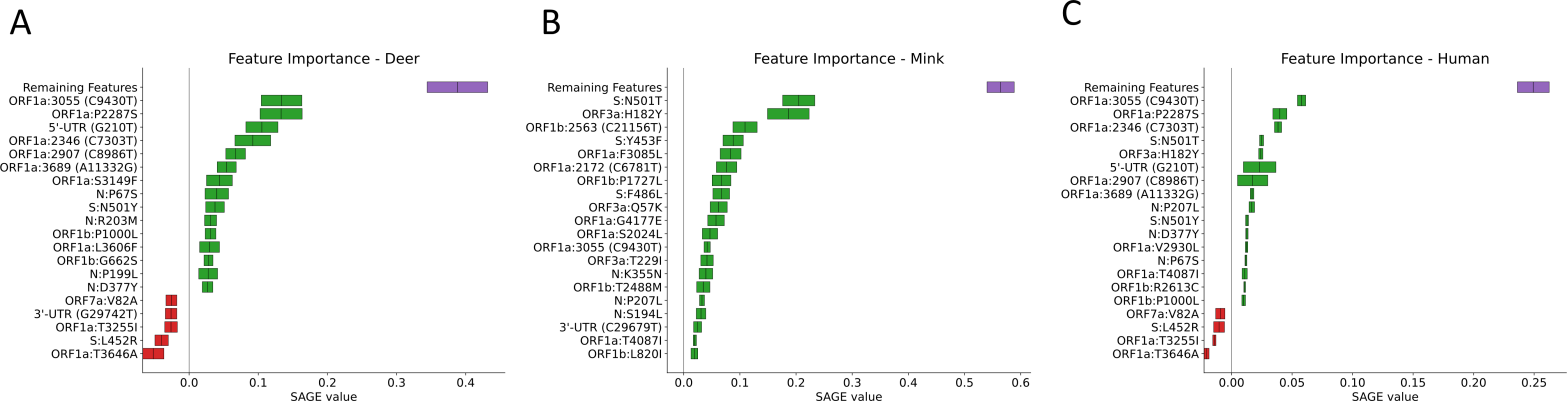
The top 20 most important features associated with each species in the “All Clades” data set. These scores are SAGE values, with higher values representing more important features. The collective importance of the remaining features is high, but unreported features found in this category have a SAGE value lower than the 20th-best actual feature. Each panel represents features important for distinguishing between deer (**A**), mink (**B**), and human (**C**) hosts. When interpreting the direction of a feature’s importance, the feature’s general presence or absence in the samples derived from the host should be determined.

When plotting the hierarchical clustering results, clear patterns of mutations distinguishing different hosts were observed in each of the data sets tested. The pattern of mutations between hosts was clearest in clade 20C (Fig. 1) with deer and mink hosts from different geographic regions often sharing few mutations in common. However, the good generalization performance we observed suggests that there exists a set of features that could be used to define each host. In clade 20C, the presence of S:N501T and ORF3a:H182Y non-synonymous mutations strongly shifted model predictions toward mink and these changes were also present in Michigan, Wisconsin, and Utah mink (in the case of S:N501T) and in Wisconsin, Utah mink (in the case of ORF3a:H182Y) (Fig. 1 and 2A). The presence of silent mutations, such as C9430T, A24232G, C13665T, and C9391T, shifts model predictions toward deer. In addition, ORF1a:S3149F appeared to be a deer biomarker since it was present in both Indiana deer and in samples from the B.1.641 lineage (Fig. 1 and 2B). The absence of mutations associated with deer and mink may also differentiate deer and mink from human samples within clade 20C (Fig. 1 and 2C). While the association between deer lineages and their characteristic mutations was not as pronounced in clade 21J (Fig. S3), two mutations (C7303T and C9430T) were important for shifting a prediction toward being a deer-derived or human-derived sample (Fig. 3A). LR used the presence of these changes to predict deer-derived strains while the absence of this change was used to predict a human-derived strain (Fig. 3; Fig. S4). A similar logic can also be used to explain the feature importance scores in Fig. 2 and 5. Interestingly, the C9403T mutation was also highly predictive in clade 20C. Finally, our analysis of the “All Clades” data set revealed a strong association between the mutation profile and clade and between the mutation profile and host species of origin (Fig. 4). The silent mutation C9430T was once again listed as one of the most important mutations associated with deer (Fig. 5A). Several mutations associated with deer from clade 21J (ORF7a:V82A, ORF7a:T120I, G29742T, ORF1a:T3255I, S:L452R, and ORF1a:T3646A) had a negative predictive value, implying that these changes are not globally predictive (Fig. 3 and 5). This makes sense since many of these are characteristic of the Delta variant and its sub-lineages (43). This likely explains why the 21J mutations were not globally predictive, even though they can be used to define an entire cluster of deer. S:N501T and ORF3a:H182Y were once again important in distinguishing mink from other sequences while human-derived sequences were generally characterized by an absence of mutations found in either deer or mink (Fig. 5B and C).

### Evolutionary analysis and predictive modeling provide complementary pictures of changes underpinning host-adaptation

**Fig 6:**
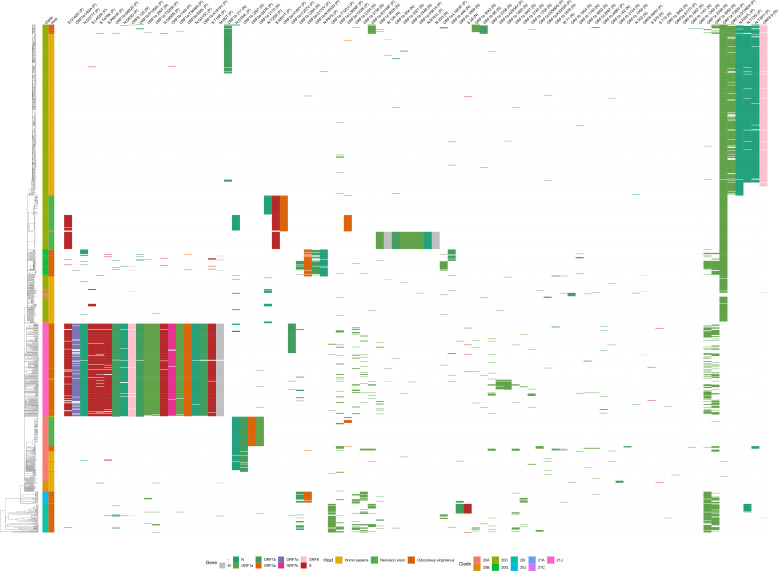
A bi-clustering of the “All Clades” data set using features from Triglav and the selection analysis. The bi-clustering shows the relationship between deer (O*docoileus virginianus*), human (*Homo sapiens*), and mink (*Neovison vison*) sequences based on their genome-wide amino acid changes relative to the reference genome, Wuhan-Hu-1 (MN908947.3). Changes in each gene are colored differently. Rows (samples) and columns (features) were clustered separately. MAFFT and IQTREE2 were used to create a phylogeny to demonstrate the relationship between samples while hierarchical clustering of features was conducted using average linkage alongside distances calculated using the Hamming metric.

**Fig 7:**
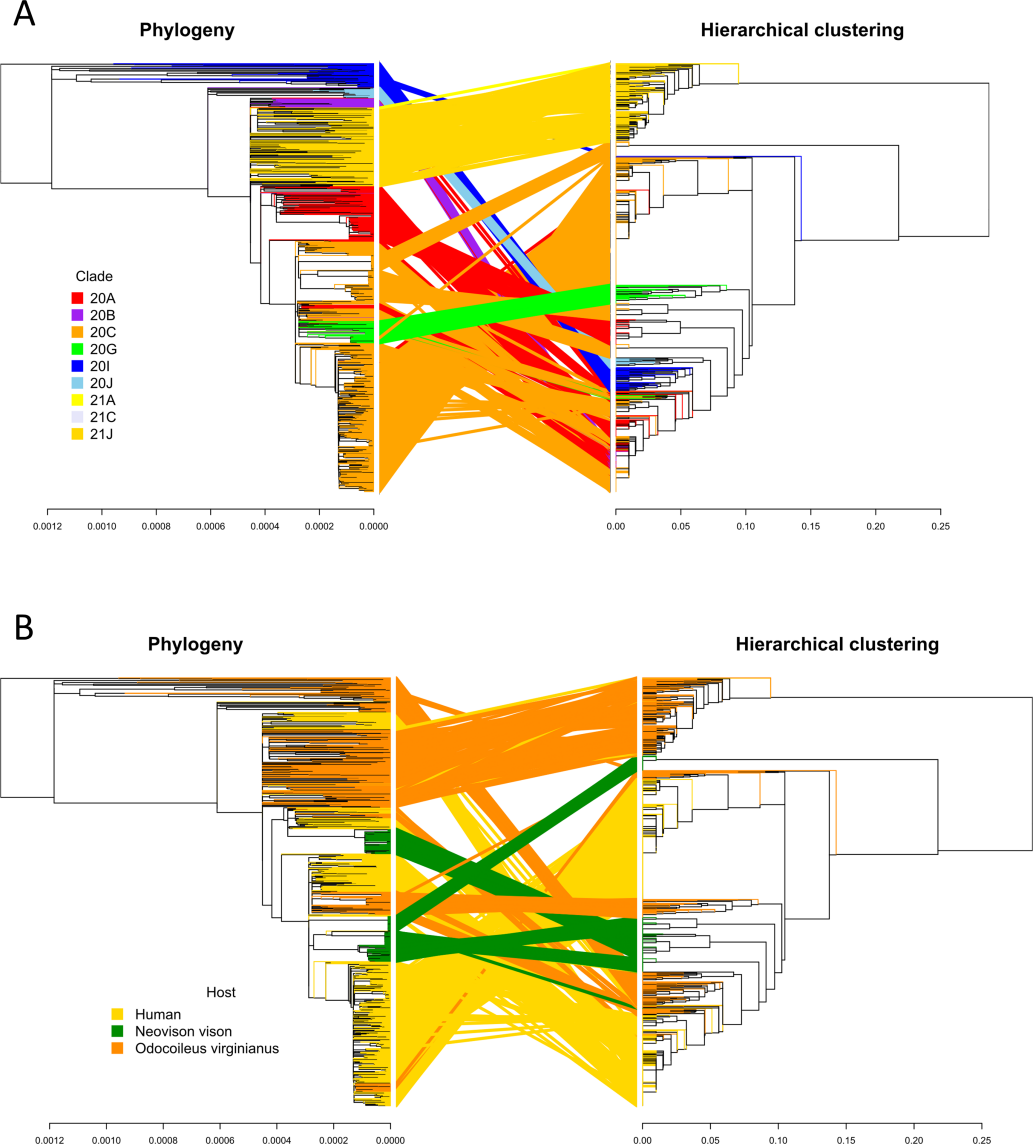
Tanglegrams between the phylogenetic tree and hierarchical clustering of the “All Clades” data. The organization of the phylogenetic time tree, created using IQTREE 2 and MAFFT multiple sequence alignments, is compared with the hierarchical clustering of samples based on their Triglav-selected features. The location of samples in both trees is colored depending on their clade (**A**) and host source (**B**).

We detected 192 amino acid changes under positive selection and 664 positions under negative selection in the “All Clades” data set for a total of 849 positions. Two hundred two unique positions were consistently detected by Triglav as predictive (*P* ≤ 0.05) across all 100 Triglav runs (Table S11). Although there was overlap between sites detected by Triglav and the selection analysis, some predictive sites were missed by one or both methods due to our choice of threshold for selecting Triglav sites and those under selection. For example, some sites in ORF8 are likely under selection since they are close to the 0.9 threshold used in this study. This is also true in reverse: some sites fall slightly under the *P* value cutoff when looking for consistently selected sites found over all of the Triglav analyses. Triglav can determine if predictive changes can be found in the 5-prime, 3-prime, and intergenic regions (Fig. 3 and 4; Table S11). This is important since the 5-prime and 3-prime ends of the genome are known to be involved in the replication of betacoronaviruses (44, 45). Eighty-eight sites are found in common between Triglav and the selection analysis (Fig. 6), and the general organization of the samples by clade and host is similar to that produced in the hierarchical clustering analysis. The organization of samples within clades 21A, 21J, and 20G was congruent between the phylogeny and the hierarchical clustering while a large fraction of samples across the other clades grouped differently or were split between different branches of the hierarchical clustering (Fig. 7A). This appears to be caused by the clustering analysis ordering samples by host source rather than the phylogenetic relationship (Fig. 7B). For example, deer sequences in clades 20I and 20C group together in one part of the hierarchical clustering since they share some changes, such as ORF1a:P2046, and mink sequences in clade 20C are split based on the unique constellation of mutations that define Michigan, Utah, and Wisconsin mink (Fig. 4 and 7B). We observe that some sites under negative selection appear to be locally (within a clade) important when discriminating host source. For example, the presence of the silent mutations C11152T, C26822T, C16329T, C10228T, and C7609T is specific for Michigan mink; the C27920T mutation is indicative of the Human III and IV clusters in clade 20C (Fig. 1 and 6), and the C20259T mutation appears to be specific for deer in clades 20G and 20I. The selection analysis also provided additional evidence supporting the global importance of the C7303T and C9430T silent mutations to predicting deer since they are present in deer across all clades (Fig. 4 to 6). Finally, when we considered sites not selected by Triglav but still under selection pressure (Fig. S4), we observed a lack of synonymous and non-synonymous mutations associated with clades 21J, 20A, 20G, and 20C. However, we did observe that different profiles of mutations could be used to describe clades 20J and 20I with one silent change (C18744T) occurring in deer in clades 21J, 20I, and 20G. Finally, both the FUBAR and SLAC analyses of the large “All Clades” data set (Tables S12 to S23) largely agree. Differences between analyses can be attributed to the algorithmic differences between methods (34).

### Tree proximity measures can be used to identify sequences containing a constellation of unique mutations related to B.1.641

**Fig 8:**
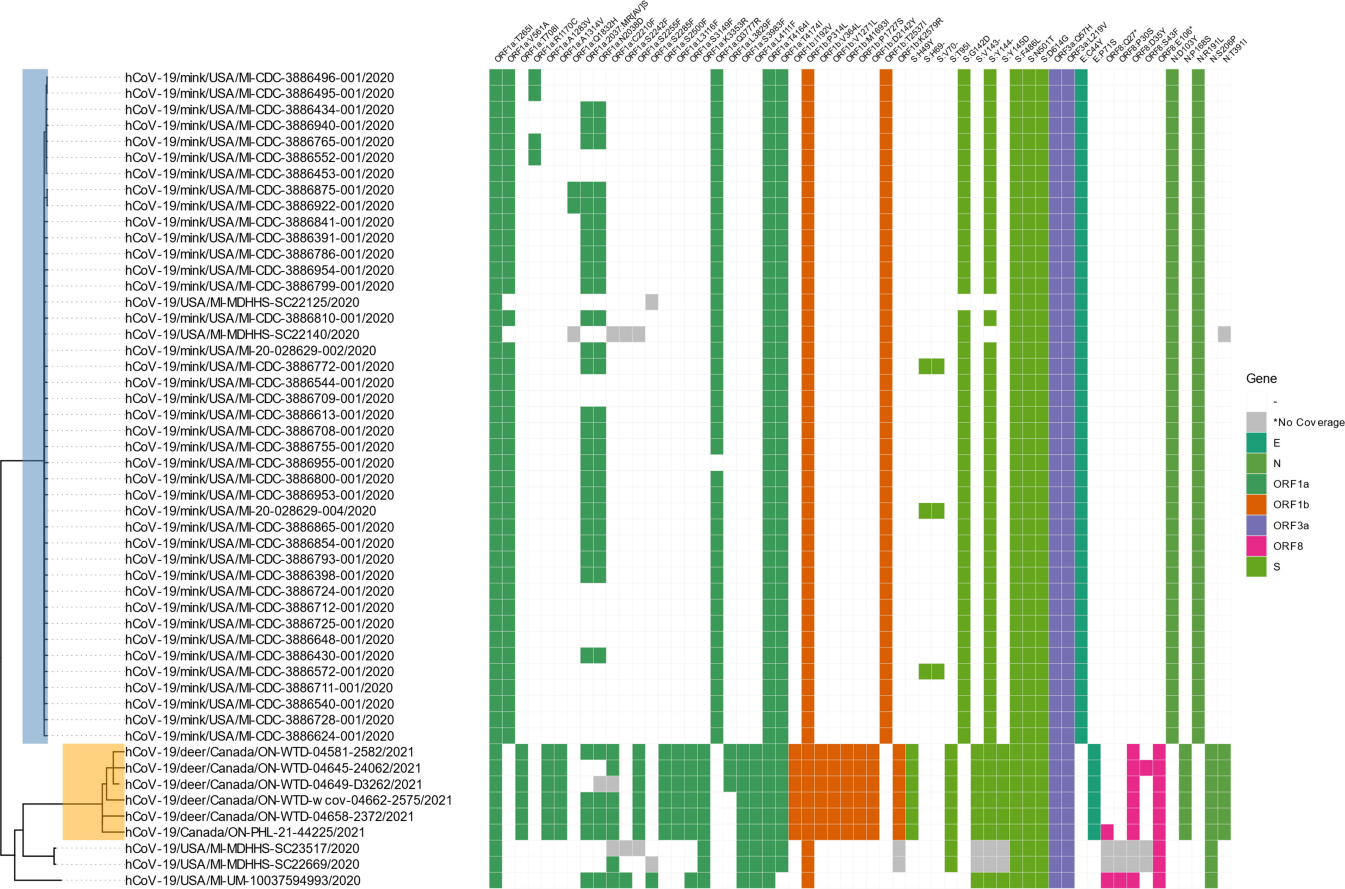
Phylogeny of the sequences identified by TreeOrdination and the nearest neighbor search. The presence and absence of amino acid mutations relative to Wuhan-Hu-1 (MN908947.3) are used to annotate each genome. Orange highlighting indicates the position of the B.1.641 lineage while the light-blue highlighting indicates the position of the related mink sequences. Amino acid mutations are colored by the corresponding gene with gray sites indicating areas that were poorly covered during sequencing.

Of particular interest is to use this approach to identify closely related sequences that may result from a spillback event from animal hosts. The unsupervised TreeOrdination analysis and nearest neighbor search allowed us to identify an additional human sequence, hCoV-19/USA/MI-UM-10037594993/2020, related to lineage B.1.641 sequences from the first reported deer-to-human transmission of SARS-CoV-2 (2). Reanalysis from the original raw Nanopore sequencing data (FAST5) was performed using Minimap2 (version 2.18), iVar (version 1.3.1), Clair3 (version 0.1.11), SnpEff (version 5.0), SnpSift (version 4.3.1t), and Bcftools (version 1.12) to confirm the mutations found in the sequence submitted to GISAID (see Additional File 2 for details on the procedure) (46–51). This reanalysis confirmed the presence of a 12-bp insertion (peptide sequence MRVS) in the ORF1a gene at position 6377. Interestingly, a similar insertion at the same position with peptide sequence MRAS was found in B.1.641 and the related Michigan mink sequences. In this analysis, the subset of features used to conduct the TreeOrdination analysis did not use the MRVS mutation since it was filtered out due to its rarity (only occurring once). Encouragingly, TreeOrdination was capable of using the presence and absence of other SNVs, SVs, and amino acids to correctly identify this sequence as close a neighbor to the query, hCoV-19/Canada/ON-PHL-21-44225/2021, the only spill back from deer to human reported to date (2). In addition to the new human sequence, the TreeOrdination analysis correctly determined that the Ontario white-tailed deer sequences in B.1.641 and several Michigan mink and human sequences were related to the query (Fig. 8). Phylogenetic analysis using IQ-TREE determined that the most recent common ancestor for this collection of related sequences likely emerged in mid-February 2020, consistent with the results reported by Pickering et al. (2). We next tested if the mutation spectrum could replicate these results. A TreeOrdination analysis using the mutation spectrum did identify the B.1.641 sequences and hCoV-19/USA/MI-UM-10037594993/2020 as related to the query. However, the Michigan mink sequences which are related to the B.1.641 lineage were not found when using the mutation spectrum (Additional File 3).

## DISCUSSION

In this work, we demonstrate that both supervised and unsupervised machine learning can successfully identify host species of origin using mutation spectrum and mutation information (SNVs, SVs, and amino acid changes). Furthermore, we demonstrate that training models using the later data greatly improves the generalization performance of the models compared with training on only the mutation spectrum. The performance of these models strongly implies that specific genetic features exist that can distinguish between viruses isolated from different hosts. We used this information to identify a human sequence from the US state of Michigan related to the recently described B.1.641 lineage from white-tailed deer in Ontario, Canada. While the evolutionary history of this lineage B.1.641 is still unclear, given that sequences from this lineage were related to sequences isolated from Michigan mink, escaped and/or intentionally released mink may have been an intermediate host in the transmission of SARS-CoV-2 to white-tailed deer in Ontario, Canada (52, 53). The analysis of minor SARS-CoV-2 variants in the sequencing data from samples collected in and around Michigan before February 2022 may provide further information about the evolution of lineage B.1.641 and its transmission history. Reanalyzing sequencing data in this manner in future pandemics could be an important practice since the allelic frequencies of characteristic mutations may fall below commonly used cutoffs. Although using the richer information from allelic frequencies (as opposed to consensus sequences) to train predictive models to recognize related strains and identify potentially adaptive changes is possible, care will need to be taken to understand which adaptations are significant since some changes will be reflective of within-host selective pressures (6, 7).

Our results showed that the predictive power of the mutation spectrum was lower than that of the underlying data set from which it was created, which could be due to the under-representation of mink and deer samples in the data set. Consistent with this hypothesis, stratified (with respect to geography) under-sampling of human-derived strains improved model generalization performance. However, training using the underlying mutation data were always preferable, as demonstrated by comparing the cross-validated generalization performances in Tables 1 and 2, which is likely due to this data set containing more useful information for classification purposes. For example, the frequency of C > U mutations in the mutation spectrum simply summarizes the number of C > U mutations found in the original data. Machine learning models, such as the ETC, may use this richer information by repeatedly partitioning the training data by selecting different C > U mutations. However, using this richer internal representation is not possible when using the mutation spectrum alone since it is a simple summary. For this reason, drawing conclusions based on evidence using the mutation spectrum should be done carefully. We must also be careful in our interpretations since we limited this analysis to three hosts. The extent to which these data sets capture the full complexity of evolutionary dynamics across all susceptible hosts remains an open question. Also, it is important to note that our use of LR, which is a linear model, limited us to identifying genomic features which are broadly responsible for predicting host source. More complex machine learning models could be used in future work to identify if co-occurrences between mutations are important for classification (54, 55). For example, it may be possible to use deep learning, such as recent advances in using transformers with tabular data, to combine the mutation spectrum and the underlying mutation data. Deep learning is an exciting avenue for investigation since the underlying representations created by the model could be used to calculate dissimilarities between samples (56). This approach can be used to generate embedding and dissimilarities by weighting different sources of information. For example, the presence or absence of SNVs, SVs, amino acid mutations and the frequency of these variants in each lineage could be used as different inputs into the model. Each of these inputs would be processed separately before being combined into a single representation. This flexibility could allow considerably more interesting analyses identifying genetic features associated with zooanthroponitic transmission. Finally, when trained using high-dimensional data (dimensions > 100), machine learning models tend to extrapolate when performing inference and prediction tasks. This occurs since samples unseen by the model almost assuredly lie outside the convex hull of the training data (57). Although the results of such an analysis are not necessarily invalid due to this behavior, they should be carefully interpreted such that the limitations imposed by the composition of the data set and multiple lines of evidence are always taken into account.

Since the generalization performance results indicated that there were genetic features strongly predictive of host species origin, Triglav was used to identify these features within the clade 20C, clade 21J, and the “All Clades” data sets (Fig. 1 to 5; Fig. S3). In mink-derived sequences, Triglav consistently identified the N501T, Y453F, and F486 Spike protein mutations as important. Site 501 was also found to be under positive selection across all clades while site 486 was just under the 0.90 probability threshold for being included as a site under positive selection. The detection of these changes by Triglav is important as they mirror the results of work studying outbreaks and adaptation in mink. For example, the changes in the spike protein detected by Triglav are associated with evasion of the immune system and antibody response, drug resistance, and spillover into ferrets (58–64). More importantly, changes such as Y453F and N501T have been experimentally confirmed as being important to successful adaptation in deer, mink, and ferrets; they enhance viral entry into cells expressing mink or ferret ACE2 (65, 66). The detection of changes such as these demonstrates that machine learning approaches, such as Triglav, can be used to search for subsets of mutations that may be involved in host adaptation.

In white-tailed deer, Triglav identified sites under negative selection, such as ORF1a:3055 (C9430T) and ORF1a:2346 (C7303T) as important for identifying deer-derived samples. The identification of these changes supports the use of Triglav as these mutations have been observed frequently in white-tailed deer while being rare in related human populations (41). We also found that the presence of changes in the 5′-UTR could be used for distinguishing deer-derived samples from those originating in mink and human hosts (Fig. 2, 3 and 5). These results mirror previously reported data which demonstrates the rarity of these mutations in human-derived isolates while providing additional support that these changes are maintained across multiple clades. Recently published evidence shows that the genome of SARS-CoV-2 is highly structured and capable of folding into specific secondary and tertiary structures (67). Different regions of the genome have also been shown to form long-range intramolecular and intermolecular interactions (68, 69). In addition, SARS-CoV-2 has been shown to use APOBEC-mediated mutations to improve the ability of viral progeny to replicate and propagate (7, 70, 71). These changes likely contribute to the observed increase in the number of silent mutations over time (72). Silent changes, such as those found here, may confer a selective advantage by optimizing codon usage and/or destabilizing RNA secondary structures ultimately resulting in improved translational efficiency in new hosts (72–74). It is also important to note that the predictive silent mutations we have identified might not be markers of cross-species transmission but could result from changes in linkage disequilibrium with amino acid changes (75). However, due to the limitations in the scope of this study, experimental validation is needed to uncover the biological role (if any) of silent mutations in host adaptation. This is necessary since mink, deer, and other mammals such as mice could become reservoirs for the virus which could result in spillovers back into humans and other animals in the future (2, 12, 76).

An important aspect of this work is the use of machine learning to identify potentially adaptive changes in SARS-CoV-2. Many of the mutations found to be predictive by Triglav for host source or under selective pressure occurred in genes other than the Spike. A substantial number of predictive sites can be found in ORF1a, ORF1b, ORF3a, M, N, and ORF8 (Table S11). Increasing evidence suggests that mutations in these genes may be linked to an increase in the frequency and type of symptoms, modulation of host immunity, and changes to immuno-dominant T-cell and B-cell epitopes (77–80, 80). For example, the S194L mutation in the nucleocapsid gene was associated with increased symptom prevalence and likely arose independently in clades 20A and 20B (77). It was also present in some deer- and mink-derived sequences in clade 20C. Given that this non-synonymous change in the nucleocapsid protein has been observed in three different lineages, it may affect virus transmissibility and host adaptation. We also observed that the presence of the R203M change in the nucleocapsid is used by models to predict white-tailed deer hosts. While the effect of this mutation is uncharacterized in deer, it and another mutation at the same location (R203K) and the D377Y change have been shown to increase viral replication and inhibit RNA-induced interferon expression in the host (81, 82). In addition, amino acid changes in the nucleocapsid protein and others, such as M and E, have been attributed to the ability of the Omicron variant to escape the immune system by decreasing the activation of natural killer cells (83). This is likely due to reduced recognition of these proteins by antibodies (83, 84). For example, ORF3a:182, N:67, and N:377 are located within putative and experimentally confirmed B-Cell and T-cell epitopes (78, 84, 85). Additional evidence also shows that non-synonymous changes such as ORF1a:T1001I can reduce the recognition of infected cells by CD8+ T cells (86). We also observed non-synonymous mutation ORF8, such as deletion variants. These were also detected in some deer hosts, and the B.1.641 lineage and these variants have been associated with increased transmissibility via the inhibition of the interferon-I pathway (80, 87, 88). Finally, the Spike protein interacts with the nucleocapsid which itself interacts with the products of the M and E genes (83). Therefore, fully uncovering the nature of the host response to infection by SARS-CoV-2 requires an understanding of the impact of mutations and any interactions that change as a result of these mutations in these genes. While the specific effect of every mutation identified in our analysis is difficult to quantify and outside the scope of this work, generally changes in the protein products encoded by these genes other than the spike protein appear to be involved in the downregulation of the class-I major histocompatibility complex (MHC-I) and inhibition of the type I interferon signaling pathway (79, 80).

The presence of the mutations identified in this study suggests a possible mechanism through which SARS-CoV-2 successfully adapts to a new host. Close contact with humans or other reservoir species increases the chances of the virus infecting non-human hosts. Upon successful infection in a new host, strong selective pressures result in an increased evolutionary rate which results in the generation of minor variants (89–92). From this population of variants, those possessing mutations that decrease the effectiveness of MHC-I, the adaptive immune system, and other components of the host’s antiviral response have a selective advantage since they result in an increased replication rate within the host (6, 83, 93). Published experimental evidence has also shown that mutations in the Spike protein occur quickly after infection and are maintained within a host and during transmission between hosts if they enhance receptor binding (7, 65). We see this in mink and white-tailed deer since the S:N501T and S:N501Y mutations enhance ACE2 binding and transmissibility, while others, such as F486L, do not substantially increase transmission among humans (3, 64, 65). Additionally, spike protein mutations in B.1.641 do not alter antigenicity in this lineage, further supporting this hypothesis (2). In addition, it is currently unknown if SARS-CoV-2 utilizes an alternative receptor in susceptible hosts. Recently, it has been shown that KREMEN1 and ASGR1 can mediate entry into cells and that the expression of these two receptors and ACE2 correlates more with susceptibility to infection than either receptor alone (94). For these reasons, it is likely that variants that enable efficient replication and immune evasion within a host come first (95). During transmission, which is a bottleneck event, variants that allow for the efficient spread between new host species are selected from the pool of intra-host variants (93). However, to verify if this hypothesis is correct, the detailed monitoring of intra-host variants and characterization of non-synonymous changes over time need to occur. It is also important to consider the effect of recombination events. While recombination has contributed to the global diversity of SARS-CoV-2, the presence of specific changes in new hosts as a result of recombination may not be adaptation due to the short existence of many viral lineages and the lack of expansion of the recombinant clades (96).

The differences we observed in the genomes of SARS-CoV-2 isolated from white-tailed deer, mink, and human hosts suggest that the cross-species transmission of the virus and its establishment in new hosts may be driven by changes in translational efficiency, antigen presentation, enhancement of receptor binding, and the antigenicity of proteins other than the spike. However, the computational nature of this work presents itself as an important limitation since we were not able to experimentally validate the role of these changes in host adaptation. This will require additional work.

### Conclusions and future work

This work demonstrates that machine learning can identify mutations capable of distinguishing SARS-CoV-2 isolated from deer, human, and mink hosts. This methodology will likely be successful in analyzing viruses isolated in other hosts and could be used to support results produced using phylogenetic approaches. Our work also underscores that a consensus using multiple lines of evidence, such as results generated using machine learning models and those produced using methods in evolutionary genomics, is necessary to begin to understand how SARS-CoV-2 and other viruses adapt to new hosts. These methods can also be used together to provide additional sources of evidence that can be used to demonstrate the relatedness of sequences. Machine learning approaches can be particularly useful if the goal is to identify genetic features related to cross-species transmission. For example, there are no assumptions involving homoplasies (multiple independent and identical changes in a nucleotide position across different lineages) since these are simply treated as features to be weighted by the model during classification. In tree-based models, such as those used in this work, if a homoplasy is predictive of a particular host, it will be used to partition samples during training. In this work, we demonstrated how both machine learning and phylogenetic analysis-based approaches were used to identify a unique SARS-CoV-2 sequence isolated from a human in Michigan and verify this sequence’s relationship to the B.1.641 lineage. Future work would involve using both phylogenetic, machine learning, and biological validation experiments as part of an automated workflow aimed at identifying and verifying the factors responsible for influencing cross-species transmission.

## Data Availability

Authors can confirm that all relevant data are included in the article and/or its supplementary information files. The code used for the project is available at https://github.com/jrudar/SCoV2_Host_Source.

## SUPPLEMENTAL MATERIAL

Supplemental file 1 spectrum.03584-23-s0001.xlsx Supplemental Tables S1 - S23.

Supplemental file 2 spectrum.03584-23-s0002.docx Reanalysis procedure for EPI_ISL_1314265.

Supplemental file 3 spectrum.03584-23-s0003.csv Nearest neighbor search results using the mutation spectrum.

Supplemental file 4 spectrum.03584-23-s0004.pdf Supplemental Figures S1 - S4.

ASM does not own the copyrights to Supplemental Material that may be linked to, or accessed through, an article. The authors have granted ASM a non-exclusive, world-wide license to publish the Supplemental Material files. Please contact the corresponding author directly for reuse.
