# Supplementary material for: Sequence signatures within the genome of SARS-CoV-2 can be used to predict host source
Source: Microbiol Spectr. 2024 Mar 4;12(4):e03584-23. doi: 10.1128/spectrum.03584-23 (PMC10986507; doi:10.1128/spectrum.03584-23)
Supplement: Supplemental file 2 — Reanalysis procedure for EPI_ISL_1314265. [file spectrum.03584-23-s0002.docx]

**Supplementary Methods – Reanalysis of EPI_ISL_1314265**

FAST5 data was basecalled and demultiplexed using Guppy (v6.3.8) using the super-high accuracy model. The basecalled reads were mapped to SARS-CoV-2 reference genome Wuhan-Hu-1 (MN908947.3) with Minimap2 (v2.18) and ARTIC V3 primers were trimmed using iVar (v1.3.1) ^31,32^. Variant calling was performed with Clair3 (version 0.1.11) using the ‘r941_prom_sup_g5014’ model and variant effect analysis was performed and summarized with SnpEff (v5.0) and SnpSift (v4.3.1t) ^33–35^. Depth-masked consensus sequences were generated using Bcftools (v1.12) and consensus sequences were generated using Bcftools depth-masked consensus sequence generation ^36^.

**References**

31. Li, H. Minimap2: pairwise alignment for nucleotide sequences. *Bioinformatics* **34**, 3094–3100 (2018).

32. Grubaugh, N. D. *et al.* An amplicon-based sequencing framework for accurately measuring intrahost virus diversity using PrimalSeq and iVar. *Genome Biology* **20**, 8 (2019).

33. Zheng, Z. *et al.* Symphonizing pileup and full-alignment for deep learning-based long-read variant calling. *Nature Computational Science* **2**, 797–803 (2022).

34. Li, H. A statistical framework for SNP calling, mutation discovery, association mapping and population genetical parameter estimation from sequencing data. *Bioinformatics* **27**, 2987–2993 (2011).

35. Cingolani, P. *et al.* Using Drosophila melanogaster as a model for genotoxic chemical mutational studies with a new program, SnpSift. *Frontiers in Genetics* **3**, (2012).

36. Li, H. *et al.* The Sequence Alignment/Map format and SAMtools. *Bioinformatics* **25**, 2078–2079 (2009).
