# Supplementary material for: Sequence signatures within the genome of SARS-CoV-2 can be used to predict host source
Source: Microbiol Spectr. 2024 Mar 4;12(4):e03584-23. doi: 10.1128/spectrum.03584-23 (PMC10986507; doi:10.1128/spectrum.03584-23)

Figure S1 – Simplified overview of the initial filtering of the dataset (A) and machine learning stage (B).

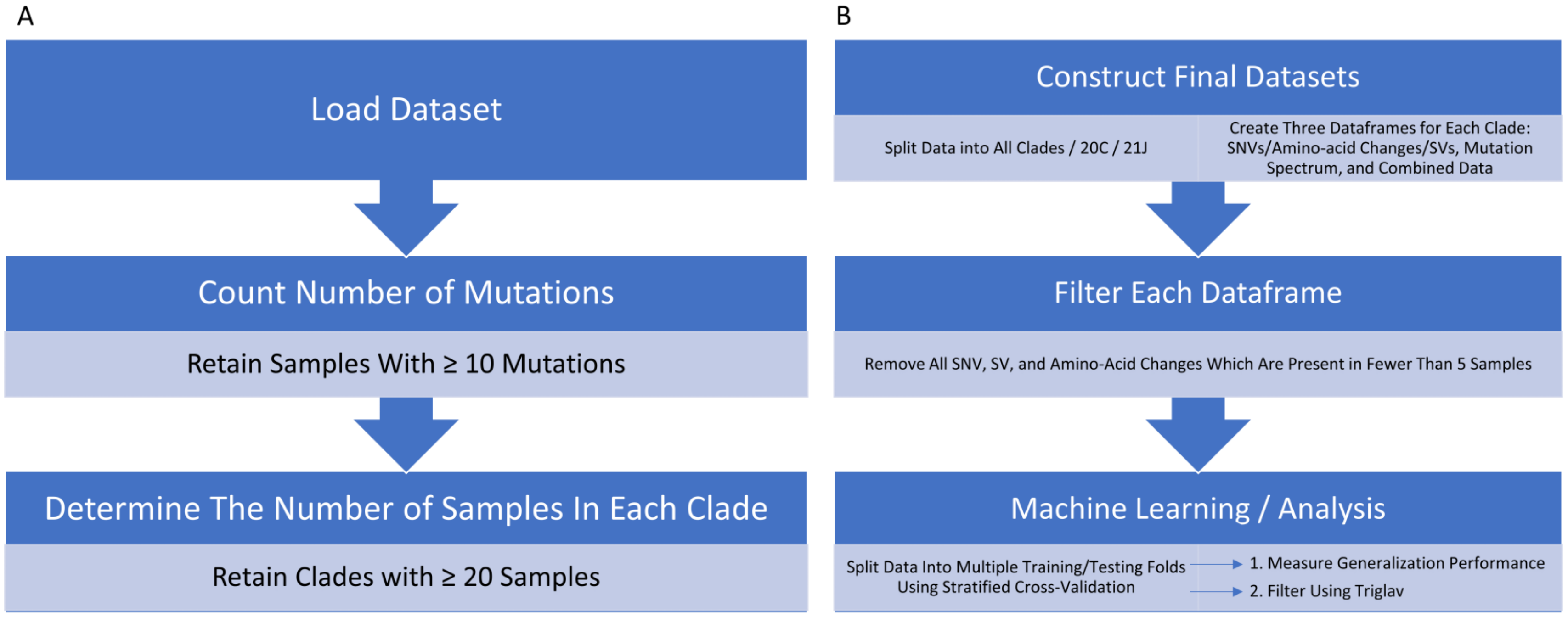

Figure S2 – Example of clustering performed by Triglav. The dendrogram visualizes feature clusters for Clade 21J. This dendrogram was produced with Triglav and unsupervised Extremely Randomized Trees. Eight hundred and forty-nine clusters are shown in this dendrogram. On average, each cluster contains 4 features.

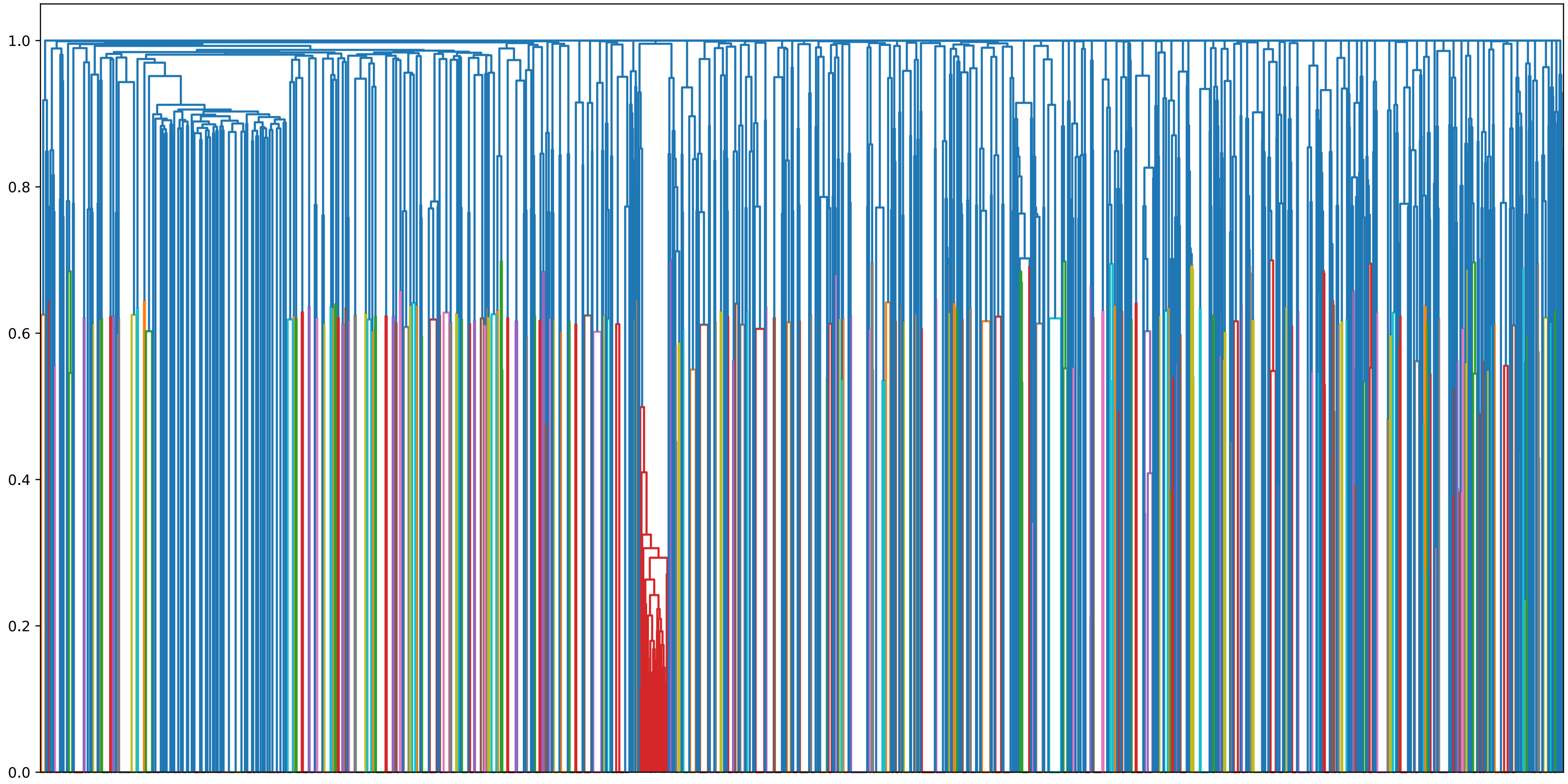

Figure S3 – A bi-clustering of clade 21J. (A) The bi-clustering shows the relationship between deer, human, and mink sequences based on their genome-wide amino-acid changes relative to the reference genome, Wuhan-Hu-1 (MN908947.3). Changes in each gene are colored differently. Rows (samples) and columns (features) were clustered separately using hierarchical clustering and average linkage with distances calculated using the Hamming metric.

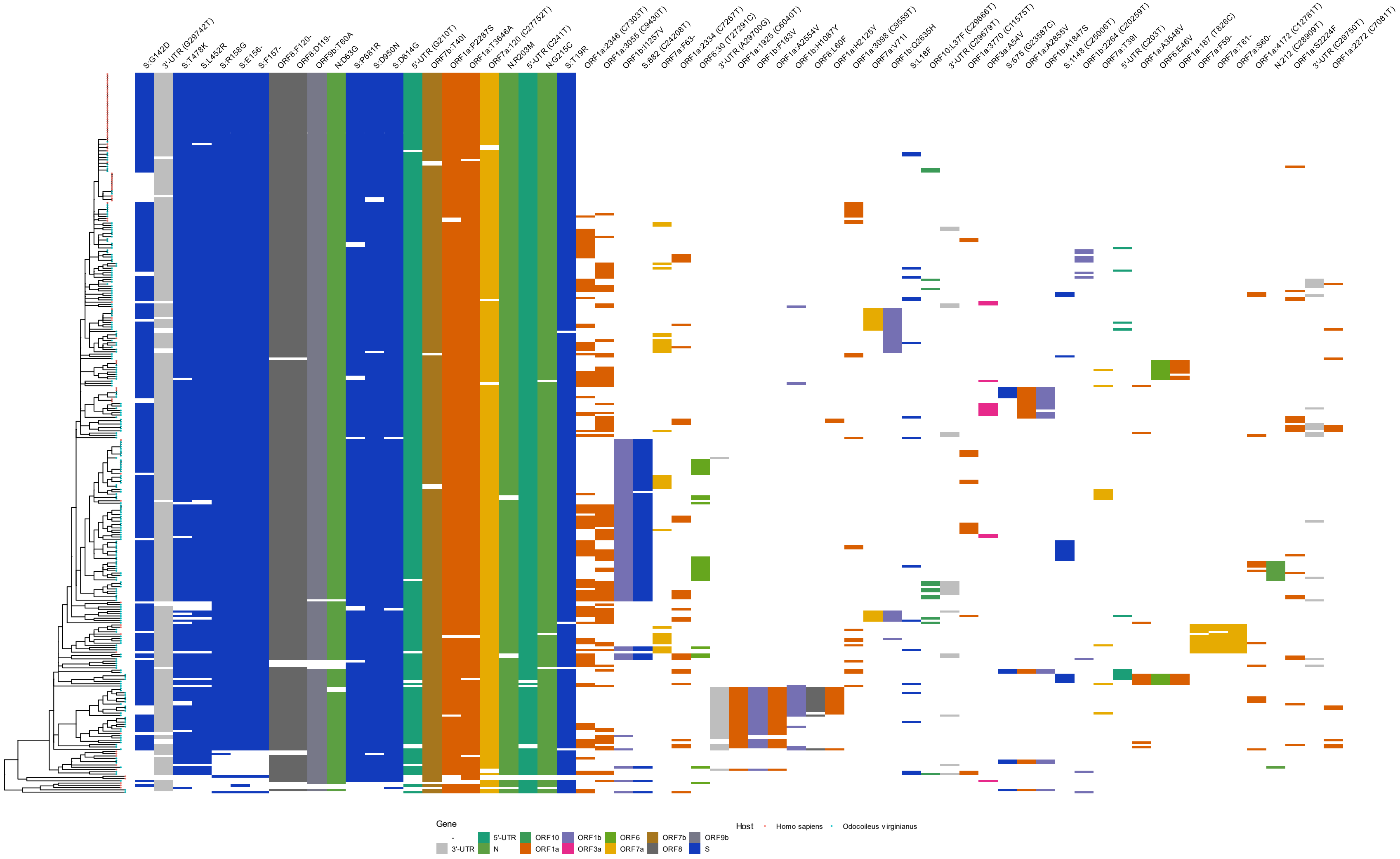

Figure S4 - A bi-clustering of the 'All Clades' dataset using features from the selection analysis not found with Triglav. (A) The bi-clustering shows the relationship between deer, human, and mink sequences based on their genome-wide amino-acid changes relative to the reference genome, Wuhan-Hu-1 (MN908947.3). Changes in each gene are colored differently. Rows (samples) and columns (features) were clustered separately. MAFFT and IQTREE2 were used to create a phylogeny to demonstrate the relationship between samples while hierarchical clustering of features was conducted using average linkage alongside distances calculated using the Hamming metric.

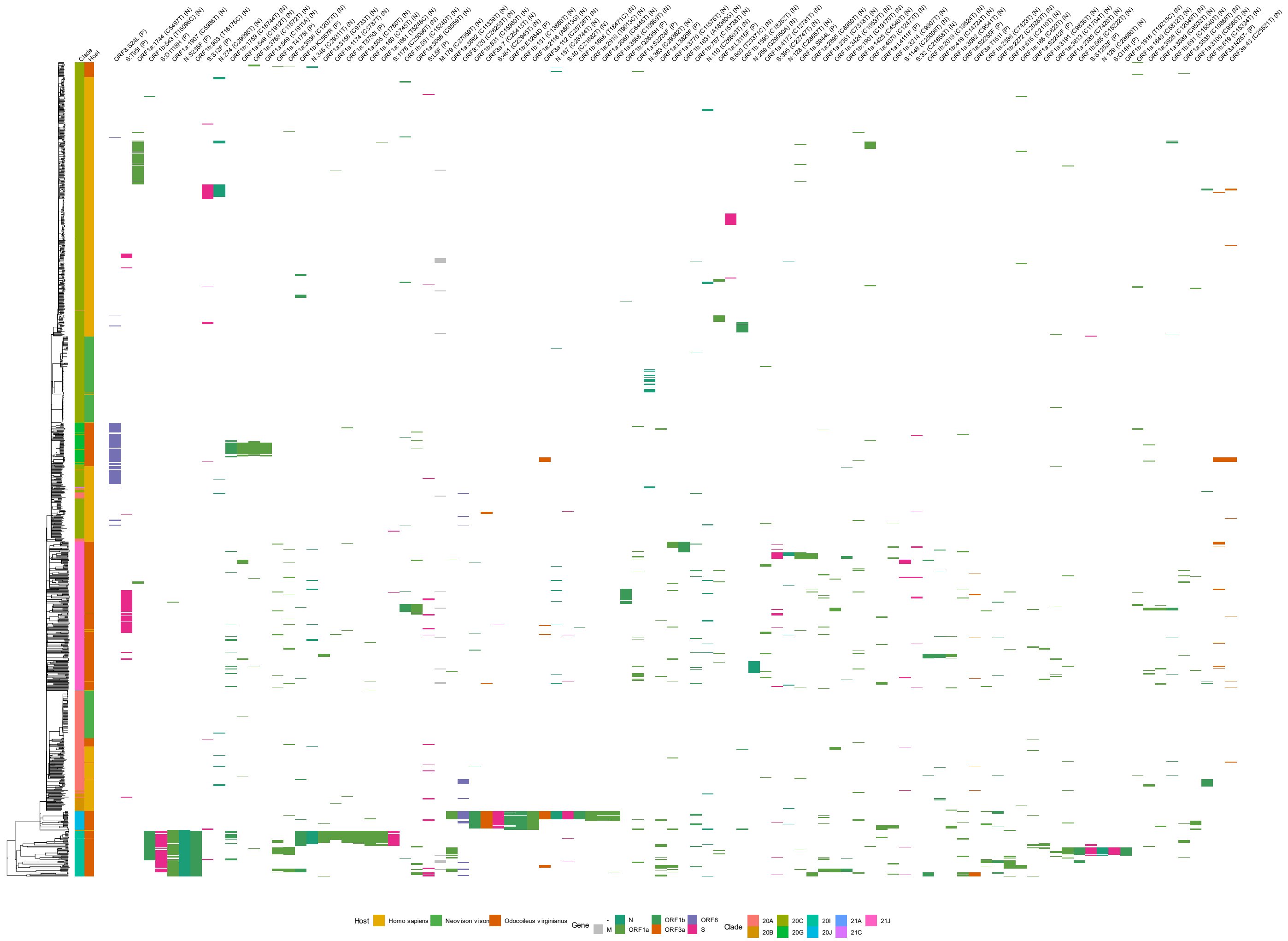

Supplement: Supplemental file 4 — Supplemental Figures S1 - S4. [file spectrum.03584-23-s0004.pdf]
